# Odd–Even Effects in the Structure and Thermal Stability of Carboxylic Acid Anchored Monolayers on Naturally Oxidized Aluminum Surface

**DOI:** 10.1021/acs.jpclett.5c00500

**Published:** 2025-03-25

**Authors:** Daria M. Cegiełka, Łukasz Bodek, Michael Zharnikov, Piotr Cyganik

**Affiliations:** ‡ Jagiellonian University, Doctoral School of Exact and Natural Sciences, Łojasiewicza 11, 30-348 Kraków, Poland; § Jagiellonian University, Faculty of Physics, Astronomy and Applied Computer Science, Smoluchowski Institute of Physics, Łojasiewicza 11, 30-348 Kraków, Poland; † Angewandte Physikalische Chemie, Universität Heidelberg, Im Neuenheimer Feld 253, D-69120 Heidelberg, Germany

## Abstract

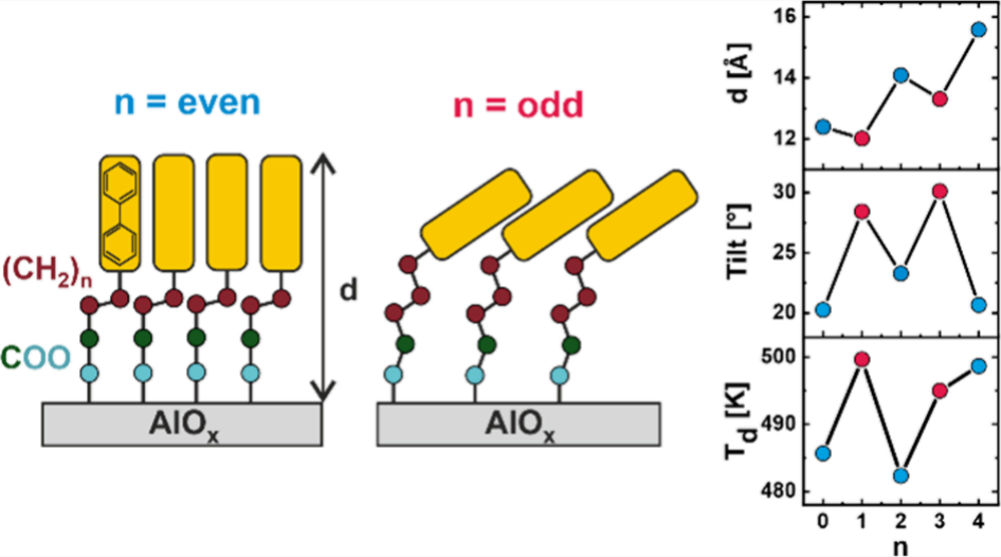

Self-assembled monolayers (SAMs) are broadly used for molecular engineering of surfaces and interfaces, which demands control over their structure and properties. An important tool in this context is the so-called odd–even effects exploiting the dependence of the SAM structure on the parity of the number of building blocks forming the backbone of SAM-building molecules. Even though these effects influence parameters crucial for SAM applications, they have been mainly studied on coinage metals (Au and Ag) until now. Here, using the series of biphenyl-substituted carboxylic acids (BPnCOO, *n* = 0–4), we show that structural odd–even behavior occurs as well on technologically relevant surface of naturally oxidized aluminum (representative of other oxide surfaces), with the *even-numbered* monolayers exhibiting higher packing density and lower molecular inclination than the *odd-numbered* analogs. Despite these structural changes, the SAM desorption energy remains nearly constant at a high value (∼1.5 eV) making BPnCOO/AlO_*x*_ a promising system for organic electronics applications.

Self-assembled monolayers^1,2^ are an established and powerful tool for tuning properties of surfaces and interfaces. They are commonly used in organic electronics,^3^ for example, to modify the electrode work function^4^ or to optimize the morphology of organic semiconductors for organic field-effect transistors (OFETs),^5−7^ as well as in biotechnology, in the role of biocompatible coatings^8,9^ or a platform for sensors.^10,11^

To match the needs of a particular application, the structure of the SAM-building molecules should be carefully designed taking into account their three crucial parts, i.e., the anchoring group, molecular backbone, and functional tail group. The anchoring group is responsible for bonding to the substrate; the functional group establishes the SAM-ambient interface, and the molecular backbone facilitates the self-assembly process. The final monolayer organization is a result of a complex interplay between the structure-building forces, such as anchoring group–substrate interactions, intermolecular interactions (chain–chain, π-π stacking), interactions between the tail groups (optionally), and steric hindrance effects (for bulky tail or side groups). The interaction with the solvent molecules during the assembly is frequently also of importance. Thus, the design of the SAM-building molecules, together with the choice of substrate and preparation conditions, determines the structure and properties of the SAMs.

Interesting phenomena, emerging from seemingly minor changes in molecular design, are the so-called odd–even effects,^12,13^ which could be observed for various hybrid, aromatic–aliphatic molecules, in which the anchoring group is linked with an unsubstituted or substituted aryl group by an aliphatic chain with variable length. For such systems, the structure of the SAM depends strongly on the parity of the number (*n*) of methylene units in the aliphatic linker, affecting parameters such as geometry and dimensions of the molecular unit cell, packing density, and molecular inclination, which all differ for *n* = odd and *n* = even. Moreover, the odd–even effects not only involve the structure of the SAMs but also include their stability toward exchange reactions,^14^ electrochemical desorption,^15,16^ and ionizing radiation.^17−19^ Thus, the odd–even effects allow for the rational design of SAMs for their specific applications related to better control over surface wettability,^20,21^ work function modification,^4^ and electron transfer and rectification^22−24^ as well as gas permeation through carbon nanomembranes.^25^ While the details of the structures and phase of the odd–even effects depend on the particular SAMs, the origin of the structural odd–even behavior is the cooperative or competitive interplay between the structural forces and so-called bending potential, related to the preferable geometry of the substrate-anchoring group-molecular chain joint.^12,26,27^ The classical example of the odd–even effects are (methyl-biphenyl)-substituted alkanethiols (CH_3_–C_6_H_4_–C_6_H_4_–(CH_2_)_n_–SH) on (111) gold and silver substrates,^27−31^ but this phenomenon has a general character and was also studied for (methyl-biphenyl)-substituted alkaneselenolates on these substrates,^32−34^ biphenyl-substituted fatty acids on Ag(111),^35^ thiols with other aryl groups,^36−38^ and even peptide-based SAMs on gold and silver.^39^

Although the odd–even effects influence the SAM properties crucial for their application in nanotechnology, until now, they were only studied on well-defined coinage metal substrates^26−39^ and semiconductor supports, such as GaAs(001).^40,41^ An interesting extension of this research would be, therefore, an analysis of possible odd–even effects on oxide surfaces that are frequently subjected to SAM engineering,^42,43^ e.g., in the context of organic electronics.^3,44^ Such a choice is particularly well-justified for a naturally oxidized aluminum surface since aluminum is an inexpensive material, commonly utilized as a gate dielectric for OFETs.^3,45−47^ Considering the functionalization of the gate dielectric in OFETs,^3,48^ the application of SAM with a hybrid aromatic–aliphatic backbone is a particularly promising strategy taking into account that (i) the aromatic tail group facilitates crystalline growth of organic semiconductor deposited on the SAM layer, improving its morphology and, as a consequence, the charge carrier mobility,^5−7^ and (ii) the introduction of the aliphatic linker enhances the isolating properties of the SAM/AlO_*x*_/Al system. Also, importantly, for organic electronics applications, the key factor is not only the SAM structure but also the thermal stability of the system because of the fabrication process performed frequently at elevated temperatures and possible overheating at the interfaces during the device operation.^49,50^

Following the above argumentation, in the current study, we show the presence of the odd–even effects for SAMs on ultrathin (∼1 nm)^51^ naturally oxidized aluminum substrates, using a series of molecules with a biphenyl tail group connected, directly or by an aliphatic linker of variable length, with the carboxylic acid anchoring group, suitable for oxidized substrates (the molecules would be further denoted as BPnCOO, *n* = 0–4). The BPnCOO SAMs were prepared using a standard immersion procedure (see Supporting Information for details). We demonstrate that the structure of these SAMs, including their packing density and molecular inclination, depends strongly on the length of the aliphatic linker, exhibiting the characteristic odd–even behavior. Our analysis also shows that this dependence does not lead, however, to prominent changes in the system’s thermal stability, introducing only rather minor odd–even variation for the molecules with short aliphatic linker.

The BPnCOO SAMs were first analyzed by XPS (Figure 1). The presence of the natural oxide on the aluminum surface is confirmed by a strong peak in the O 1s spectra (Figure 1c) and the presence of two components in the Al 2p spectra (Figure 1b), corresponding to metallic (**1**; ∼72.7 eV) and oxidized (**2**; ∼75.4 eV) aluminum. The C 1s signal comprises two peaks (Figure 1a). The main component at 285.7–285.9 eV **(1)** is an overlap of signals originating from the biphenyl moiety and aliphatic linker (SAM matrix), while the small peak at ∼290.0 eV **(2)** is related to the carboxylic group. The presence of a single peak originating from the latter group, at the position characteristic for the O–C=O bond,^52,53^ indicates a uniform bonding mode for all molecules in the SAM, most likely as a monodentate configuration suggested previously in literature for carboxylic acids on aluminum.^54,55^ We note that the main C 1s peak is electrostatically shifted^56^ by ∼1.2 eV, compared to the analogous thiolate-anchored molecules (CH_3_–C_6_H_4_–C_6_H_4_–(CH_2_)_2_–SH) on gold (284.5 eV)^57^ due to the presence of an inherent dipole layer on the aluminum surface.^51^ Most importantly, the intensities of the XPS peaks for the BPnCOO series change consistently with *n*, exhibiting clear odd–even behavior (Figure 1d–f). While the geometrical length and number of the carbon atoms in the molecule rise progressively with increasing *n*, the intensity of the C 1s main peak is systematically lower for *n* = odd compared to *n* = even. This behavior is accompanied by the opposite, “up-down” variation of the substrate signals (Al 2p and O 1s), suggesting a systematic variation of the SAM thickness. Indeed, this parameter, estimated based on the C 1s intensity for the BPnCOO SAMs relative to the reference system of hexadecanethiol (HDT) on gold,^51,58^ shows a distinct odd–even behavior (Figure 1g and Table 1), implying that the BPnCOO molecules with *n* = even form thicker and more densely packed monolayers than those with *n* = odd.

**Figure 1:**
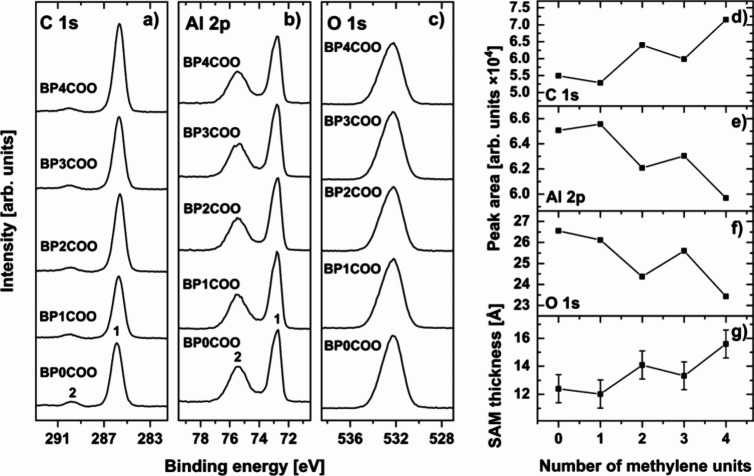
Summary of the XPS data for the BPnCOO series (*n* = 0–4). (a–c) C 1s (a), Al 2p (b), and O 1s (c) spectra. The individual components of the C 1s and Al 2p spectra are marked by numbers (see the text for details). (d–g) The intensities of the main C 1s (d), Al 2p (e), and O 1s (f) peaks and the estimated SAM thickness as functions of *n* (g).

**Table 1:** Summary of the parameters measured for the BPnCOO/AlO_*x*_ series

BPnCOO/AlO_*x*_	***n*** = 0	***n*** = 1	***n*** = 2	***n*** = 3	***n*** = 4
**SAM thickness [Å]**	12.4(±1)	12(±1)	14.1(±1)	13.3(±1)	15.6(±1)
**Tilt angle of π**_1_* TDM (ρ) [°]	72.9(±3)	66.2(±3)	70.4(±3)	64.8(±3)	72.6(±3)
**Tilt angle of SAM (φ)**^a^**[°]**	20.3(±3)	28.4(±3)	23.3(±3)	30.1(±3)	20.7(±3)
**Desorption temperature [K]**	486(±8)	500(±11)	482(±11)	495(±6)	499(±19)
**Desorption energy [eV]**	1.48(±0.03)	1.52(±0.03)	1.47(±0.03)	1.51(±0.02)	1.52(±0.06)

a Assuming the twist angle of 32°.

It is worth noting that the “phase” of the odd–even changes in the XPS signals for BPnCOO/AlO_*x*_ is the same as reported earlier for BPnCOO/Ag, where thicker and better-ordered films are also observed for *n* = even.^35^ Note also that, generally, the phase of the odd–even effects originates from the specific geometry of the anchoring group-centered joint on a particular substrate, which is “transferred” to the aromatic tail group by the aliphatic linker, having most frequently all-trans conformation.^12^ The same phase for aluminum and silver suggests, therefore, that both monodentate bonding configuration on aluminum^54,55^ and bidentate bonding configuration on silver^35,57^ ensure similar geometric arrangements for the molecular backbones. Specifically, it suggests that the bond between the carboxylic group and adjacent carbon atom (in the aliphatic linker or biphenyl moiety) in BPnCOO/AlO_*x*_ is almost perpendicular to the aluminum surface, as it occurs for the BPnCOO SAMs on silver.

The BPnCOO/AlO_*x*_ series was also studied by NEXAFS spectroscopy (Figure 2) to assess the influence of *n* on the molecular orientation. The respective C K-edge spectra, collected at an X-ray incidence angle of 55° (Figure 2a) and unaffected therefore by molecular orientation,^59^ exhibit the characteristic absorption structure of phenyl rings, with the most prominent π_1_* resonance **(1)** at ∼285.0 eV.^29,32,60,61^ This resonance is accompanied by the π_COO_* resonance of the carboxylic group at ∼288.5 eV^51,62^ overlapping with the π_2_* resonance from the biphenyl moiety at ∼288.8 eV.^60^ Moreover, the small feature **(2)** at ∼287.5 eV could be assigned to the Rydberg resonance.^61,63^

**Figure 2:**
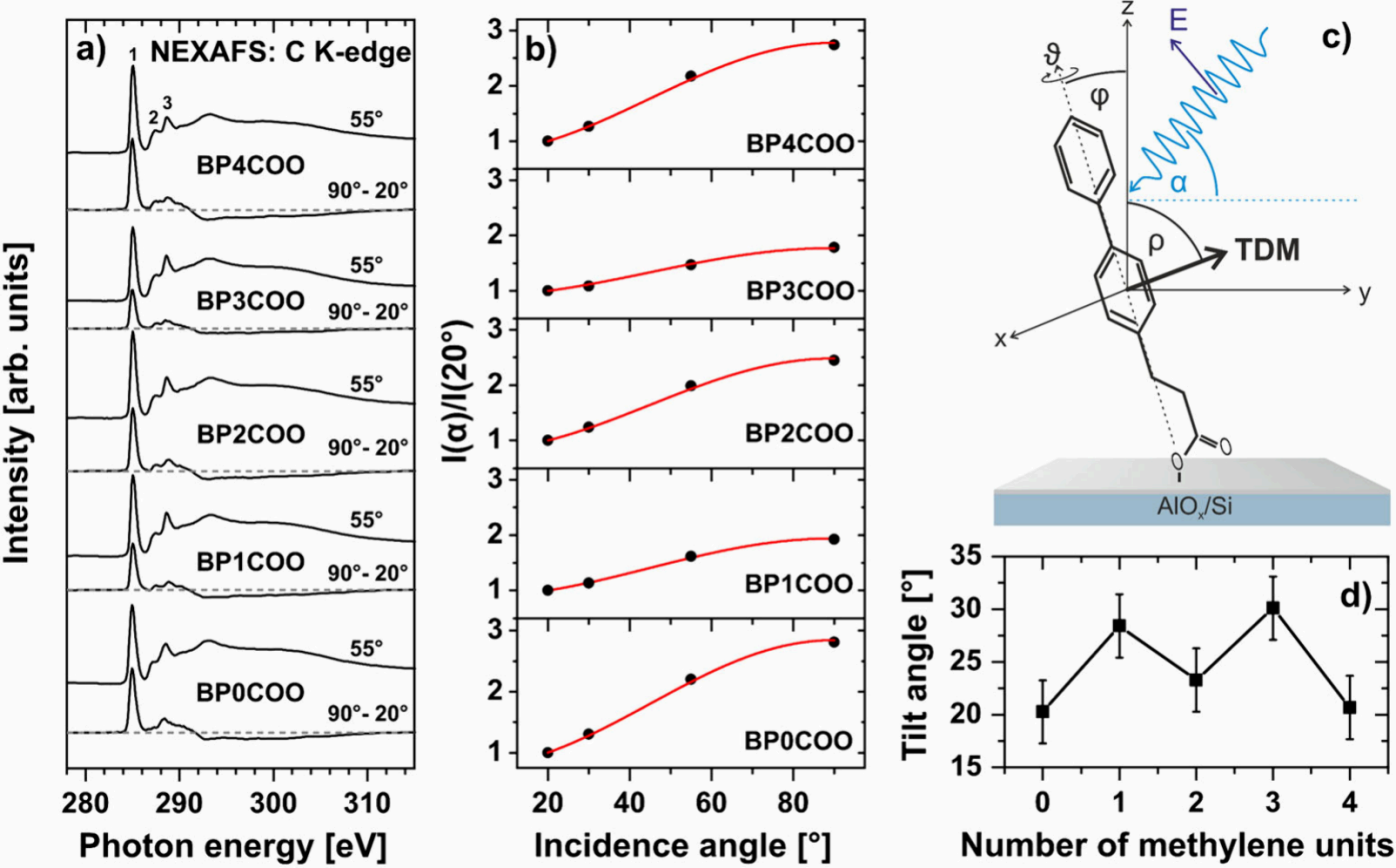
Summary of the NEXAFS spectroscopy data for the BPnCOO series, *n* = 0–4. (a) C K-edge spectra collected at an X-ray incidence angle (α) of 55° and the difference between the spectra recorded at the normal (α = 90°) and grazing (α = 20°) incidence geometry. The horizontal dashed lines indicate the zero level for the difference spectra, and the individual resonances are marked by numbers (see text for details). (b) The ratio of the π_1_* resonance intensities as a function of X-ray incidence angle (black circles) with the best fits according to eq 1(red solid lines). (c) The schematic representation of the experimental geometry and the definition of the angles defining the molecular orientation. (d) The dependence of the average molecular tilt angle (φ) on *n*.

The intensity of the π_1_* resonance (and the other resonances as well) depends on the X-ray incidence angle (α), as shown by the difference spectra (Figure 2a) calculated by subtraction of the spectra recorded at the grazing incidence geometry (α = 20°) from those measured at the normal incidence (α = 90°). Such a phenomenon is known as linear dichroism^59^ and is an indicator of molecular orientation. As π_1_* is a vector orbital with the transition dipole moment (TDM) perpendicular to the plane of the phenyl ring (Figure 2c), the positive sign of linear dichroism (the highest intensity at normal incidence) implies an upright orientation of the BPnCOO molecules in the SAMs. However, the absolute value of the dichroism for the BPnCOO series is higher for *n* = even than for *n* = odd, suggesting differences in the molecular inclination.

These differences were addressed by a numerical evaluation of the NEXAFS data, relying on the intensity of the π_1_* resonance. The intensity of a resonance associated with a vector-like orbital is generally expressed as
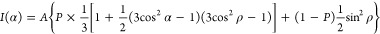
1where *A* is an orbital-specific constant, α is an incidence angle of the X-rays, *P* is the polarization factor, and ρ is an angle between the TDM of the π_1_* resonance and the substrate normal (Figure 2c).^59^ The values of ρ for the BPnCOO series, obtained by fitting the experimental data to eq 1 (Figure 2b), are presented in Table 1 (see Supporting Information for details). These values can be further used to derive the tilt angle (φ) of the molecules (Figure 2c) assuming the twist angle (ϑ) of 32° for the biphenyl moieties (as in the bulk crystals)^27,61^ and employing the equation^27,60^
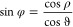
2

The calculated average molecular tilt angles for BPnCOO/AlO_*x*_ are presented in Figure 2d and Table 1. These values exhibit a clear odd–even behavior with a larger tilt for *n* = odd (φ ≈ 29°) and a smaller tilt for *n* = even (φ ≈ 21°). This behavior agrees well with the results of the XPS analysis since the smaller molecular tilt is associated with a larger film thickness and higher packing density, derived for *n* = even.

We note at this point that the phase and magnitude of the odd–even effect in the molecular orientation for BPnCOO/AlO_*x*_ are similar to those observed for the (methyl-biphenyl)-substituted alkanethiols (BPnS)^27^ and selenols (BPnSe)^32^ on silver. Thiols and selenols prefer to bond to silver in *sp*-like configuration, which gives the Ag–S(Se)-C angle of ∼180°, defining the phase of the odd–even behavior. Thus, the resemblance between the molecular orientation of biphenyl-substituted thiols/selenols on silver and carboxylic acids on aluminum is yet another argument suggesting the close to perpendicular orientation of the first C–C bond (starting from the substrate; see Figure 3) in the BPnCOO/AlO_*x*_ series. This orientation is favorable for dense molecular packing at *n* = even (Figure 3). However, assuming the fixed and exactly perpendicular orientation of this bond and all-trans conformation of the aliphatic linker, one would get a much stronger inclination of the biphenyl units for *n* = odd compared to the recorded values (Table 1). This would then lead to a drastic reduction in the surface coverage, which is energetically unfavorable and has yet not been recorded for any system exhibiting the odd–even effects.^12^ The outcome of this dilemma is a compromise between the preferred molecular geometry, driven by the bending potential, and the structural forces, favoring a high packing density, resulting in optimization of the AlO_*x*_–OOC-C angle (marked as ω’ in Figure 3) to keep the area per molecule as small as possible for canted orientation of biphenyl moiety for *n* = odd.

**Figure 3:**
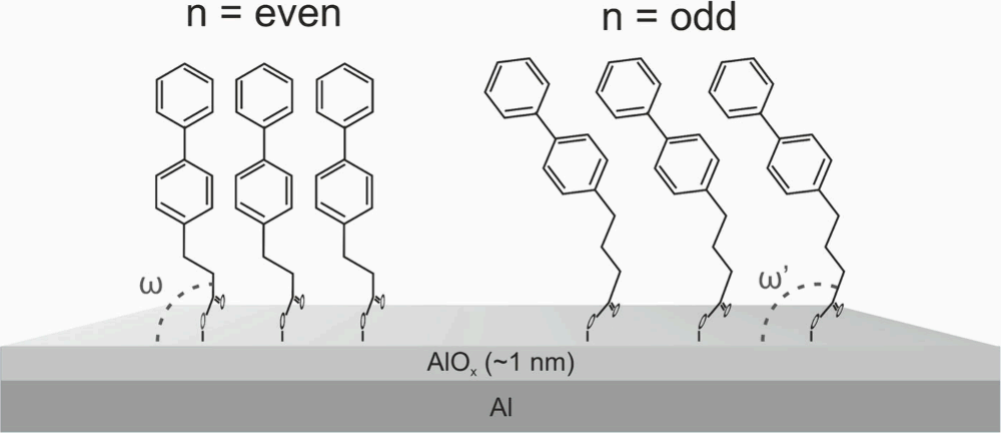
Schematic representation of the odd–even effects for the BPnCOO SAMs on the naturally oxidized aluminum surface. The parameter defining the bending potential associated with the AlO_*x*_–OOC-C joint is the angle (ω or ω’) between the respective C–C bond and the substrate.

Knowing the impact of the length of the aliphatic linker on the monolayer structure, temperature-programmed XPS (TP-XPS) measurements were conducted to assess the influence of these structural changes on the thermal stability of the system. The obtained desorption profiles are presented in Figures 4 and S2. The process was monitored using the intensity of the main C 1s peak, associated with the molecular backbone (Figure 4a), and the total Al 2p signal originating from the substrate (Figure 4c). According to these data (see also Supporting Information for details), the C 1s signal progressively decreased and the Al 2p signal increased during heating, which indicates a progressive thickness reduction mediated by the desorption of the molecules and their fragments. Moreover, the C 1s signal associated with the carboxylic group decreases similarly to the main C 1s peak (Figure S3), which indicates that desorption proceeds via cleavage of the chemical bond between the carboxylic group and the substrate or within this group. It is worth noting that the desorption profiles for BPnCOO/AlO_*x*_ are rather broad (∼80 K), which can be related to the intrinsically inhomogeneous nature of the substrate and the progressive change in the properties of the SAMs in the course of the temperature-induced desorption.

**Figure 4:**
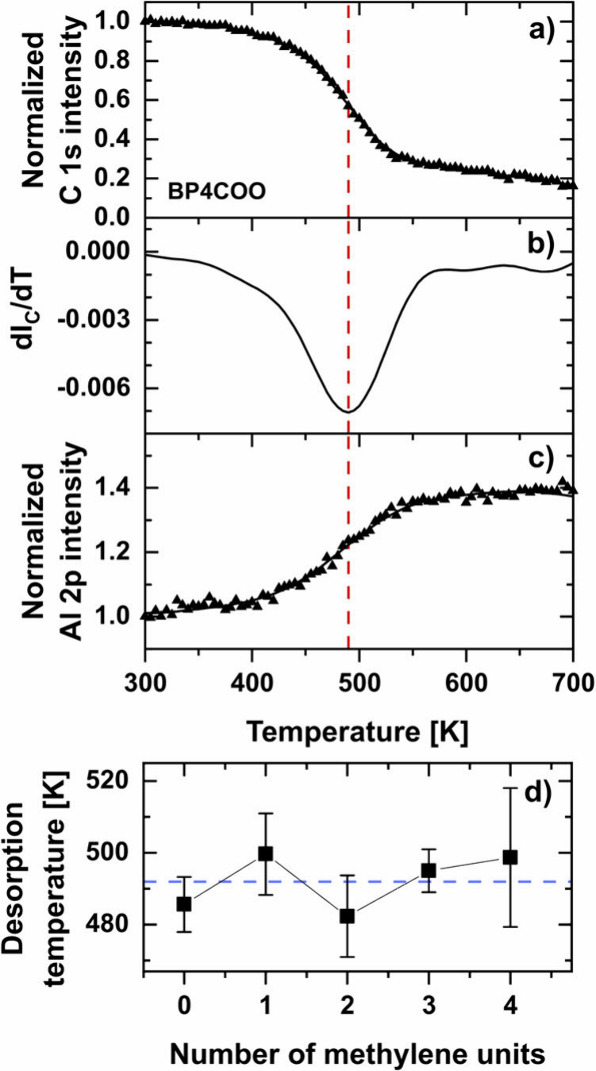
Representative TP-XPS data for BP4COO/AlO_*x*_ (all data are presented in Supporting Information). (a) Temperature profile for the C 1s intensity associated with the molecular backbone. (b) Smoothed derivative of this profile used for determining the desorption temperature. (c) Temperature profile for the Al 2p intensity. (d) Dependence of the desorption temperature for BPnCOO/AlO_*x*_ on *n*. The desorption temperature was calculated as a mean of three measurements. The error bars represent the maximal difference between a particular value in the series and the mean value. The blue horizontal dashed line indicates the mean of the desorption temperature values, irrespective of the SAM type, and serves as a guide to the eye.

Interestingly, not only the intensity of the main C 1s peak but also its binding energy (BE) changes during the heating, increasing, in particular, by ∼0.35 eV in the range of 300–400 K (Figure S4a) where no significant changes in the C 1s intensity are visible (in contrast to aliphatic carboxylic acids on aluminum, for which a similar effect was reported^58^). The BE change is also not connected to the modification of the substrate, including its inherent dipole layer, as the metal/oxide ratio for the Al 2p signal remains unchanged in this temperature region (Figure S4b). A possible reason for the shift might be, therefore, a temperature-induced change in the bonding configuration of the anchoring carboxylic group^64^ accompanied by a charge density rearrangement causing a change in the electrostatic shift. We think, however, that the bonding configuration most likely stays monodentate as no additional carbon component, suggesting a coexistence of monodentate and bidentate configurations, was observed during the temperature ramping.

To quantitatively assess the BPnCOO/AlO_*x*_ desorption temperature, the first derivative of the C 1s profile was calculated (Figure 4b), and its minimum was taken as the sample desorption temperature (additional details of the temperature profile analysis are available in Supporting Information). The mean desorption temperatures for the BPnCOO/AlO_*x*_ series, derived from three independent TP-XPS measurements for each *n*, are presented in Figure 4d and Table 1. The average (over the series) value of the desorption temperature is comparably high (∼492 K), which shows the potential of the BPnCOO SAMs for application-related engineering of AlO_*x*_. In addition, this parameter exhibits a small, odd–even variation for *n* = 0–3, followed by saturation for the longer linkers. Surprisingly, these are the more tilted and less densely packed odd-numbered SAMs that exhibit higher thermal stability than the monolayers with *n* = even. Note that the bond to the substrate (AlO_*x*_–O) in the latter systems is less strained than in the former ones, because of the cooperative interplay of the structural forces and bending potential (in contrast to the competitive interplay for *n* = odd), but this is not necessarily the bond to the substrate that governs the thermal stability of a SAM. In many cases, this is the adjacent bond, representing the so-called “weak link” in the system. Relevant examples are thiols and selenols on gold^65−67^ and silver,^67−69^ where stronger bonding to the substrate via the anchoring group (Se–Au(Ag) > S–Au(Ag)) can weaken the adjacent bond (Se–C < S–C) and significantly decrease the thermal stability of the whole system. Such oscillations in the stability of consecutive bonds^66,70^ might also be the reason for the lower thermal stability of the even-numbered BPnCOO/AlO_*x*_ as we can assume that the larger strength of the AlO_*x*_–O bond will lead to a weakening of the neighboring O–C bond which becomes the “weak link” of the system determining its lower thermal stability compared to the odd-numbered SAMs. The effect should, however, be small considering the small extent of the odd–even variation of the desorption temperature (∼15 K) compared to the ∼80 K width of the TP-XPS profiles for BPnCOO/AlO_*x*_.

The use of the TP-XPS protocol, with a constant heating rate (∼3.6 K/min), allows us to calculate the desorption energy, using the Redhead formula^71^ (with the typical frequency factor of 10^13^ Hz, applied also in previous analyses^67,68,72−74^). The obtained results, representing the first experimental determination of the desorption energies for SAMs on aluminum (to the best of our knowledge), are summarized in Table 1. Accordingly, the desorption energies for BPnCOO/AlO_*x*_ are in the range of 1.47 – 1.52 eV, which is significantly higher than those of the archetypical alkanethiols on gold (∼1.32 eV)^72^ and silver (∼1.42 eV)^68,75^ and those of aromatic, naphthalene-based, thiols and selenols on gold (1.35 and 1.2 eV respectively).^67^ At the same time, these values are very similar to those for carboxylic acids with a naphthalene backbone on silver (1.52 eV).^68^

In conclusion, our results demonstrate that odd–even effects in the structure of hybrid, aromatic–aliphatic SAMs do exist not only on well-defined surfaces of coinage metals and semiconductors but also on complex and inhomogeneous surfaces of oxides, with naturally oxidized aluminum being a representative test system. By the example of BPnCOO/AlO_x_, we show that the length of the aliphatic linker affects strongly the molecular packing density and inclination, with densely packed and less inclined SAMs for *n* = even than *n* = odd. Moreover, the phase of the odd–even effects gives us insight into the geometry of the bonding configuration of carboxylic acids on aluminum. Finally, the presented thermal analysis shows that the observed structural odd–even changes at the molecule–substrate interface induce rather small odd–even changes in the relatively high desorption energy of the system, which is close to ∼1.5 eV for short aliphatic linkers and seems to saturate quickly with its increasing length. Thus, considering the high structural quality, tunable packing density, and thermal stability, we believe that biphenyl-substituted carboxylic acids on aluminum are promising systems for applications in organic electronics. An additional option is the decoration of the BPnCOO molecules with specific tail groups, promoting the growth of an organic semiconductor and/or affecting positively the charge carrier density in the adjacent active layer, e.g., by mitigation of interfacial defects. Also, these molecules can be possibly applied to other application-relevant oxide substrates including naturally oxidized titanium and tantalum. This, however, will require further investigations, which will hopefully be stimulated by the presented results.

## Supporting Information Available

Experimental details on (1) sample preparation, (2) XPS measurements, (3) NEXAFS spectroscopy measurements; details of NEXAFS spectroscopy analysis; details of TP-XPS analysis and TP-XPS results for all samples studied (PDF)
Transparent Peer Review report available (PDF)

## Abbreviations

SAM: self-assembled monolayers
XPS: X-ray photoelectron spectroscopy
TP-XPS: temperature-programmed X-ray photoelectron spectroscopy
NEXAFS: near-edge X-ray absorption fine structure
BPnCOO: C_6_H_5_–C_6_H_4_–(CH_2_)_n_–COO
