# Supplementary material for: Odd–Even Effects in the Structure and Thermal Stability of Carboxylic Acid Anchored Monolayers on Naturally Oxidized Aluminum Surface
Source: J Phys Chem Lett. 2025 Mar 25;16(13):3343–50. doi: 10.1021/acs.jpclett.5c00500 (PMC11973916; doi:10.1021/acs.jpclett.5c00500)
Supplement: Supplementary file 1 — jz5c00500_si_001.pdf [file jz5c00500_si_001.pdf]

Supporting Information for:

Odd-Even Effects in the Structure and Thermal Stability of Carboxylic Acid Anchored Monolayers on Naturally Oxidized Aluminum Surface

Daria M. Cegiełka,[‡ §]* *Łukasz Bodek,*[§] *Michael Zharnikov,*[†] *and Piotr Cyganik*[§]*

[‡] Jagiellonian University, Doctoral School of Exact and Natural Sciences, Łojasiewicza 11, 30-348 Kraków, Poland

[§] Jagiellonian University, Faculty of Physics, Astronomy and Applied Computer Science, Smoluchowski Institute of Physics, Łojasiewicza 11, 30-348 Kraków, Poland

[†] Angewandte Physikalische Chemie, Universität Heidelberg, Im Neuenheimer Feld 253, D-69120 Heidelberg, Germany

* Author to whom correspondence should be addressed.

KEYWORDS: aluminum, carboxylic acids, odd-even effect, XPS, NEXAFS spectroscopy, thermal stability

1. Materials and methods

1.1 **Sample preparation.** Thin (~100 nm) aluminum (purity 99.999%, Kurt J. Lesker) or gold (purity 99.99%, Testbourne) layer was evaporated on silicon wafer (110) (Si-Mat Silicon Materials, Gemarny) with a rate of ~1-2 nm/s for aluminum and ~ 0.1 nm/s for gold. For the evaporation of gold on silicon, ~ 4 nm layer of chromium was used to improve the adhesion between metal and silicon. For the thermal stability experiments, the respective metal was evaporated on freshly cleaved mica sheets (V1 grade, Ted Pella, USA). Mica substrates were annealed overnight, and the evaporation process was held at an elevated temperature of ~360°C for aluminum. Before further proceeding, the substrate was cooled to room temperature. After the evaporation process, the aluminum substrates were transferred to a glovebox (MBraun) in an argon atmosphere, to better control the aluminum oxidation process and to limit the humidity during the SAMs formation, while the processing of gold was held in air. Regardless of the transfer procedure, the aluminum was naturally covered by a thin oxide layer and such a system will be further denoted as aluminum. The SAMs were prepared by immersion of freshly prepared aluminum substrates in 1mM solutions of respective BPnCOO molecules in isopropyl alcohol (PureLand, Poland, 99.9%) at room temperature for 24h. The BPnCOO series precursors – 4-Biphenylcarboxylic acid (Alfa Aesar, 98%), 4-Biphenylylacetic acid (Alfa Aesar, 98%), 3-(4-Biphenylyl)propionic acid (Alfa Aesar, 98%), 4-(4-Biphenylyl)butyric acid (Wako FUJIFILM, 94%), and 5-(4-Biphenylyl)valeric acid (Enamine, 95%) - were purchased commercially and used without any further purification. As a reference system, the 1-hexadecanethiol (Alfa

Aesar, 97%) SAM on gold was prepared by immersion in ethanol solution for 24h at room temperature.

1.2 **X-ray photoelectron spectroscopy (XPS) measurements.** XPS experiments were carried out using an ESCALAB™ QXi Microprobe workstation manufactured by ThermoFisher Scientific. The system is equipped with a 180° double-focusing, bi-polar hemispherical analyzer and monochromate, microfocused Al Kα (E = 1486.6 eV) X-ray source. The measurements were taken in an ultrahigh vacuum (UHV) with a base pressure below 5 x 10^{-10} mbar. Spectra were collected at normal emission geometry with 0.1 eV energy step using 650μm X-ray spot, after the sample position was carefully adjusted. The Au $4f_{7/2}$ peak at 84.0 eV was used to calibrate the binding energy scale and its full width at half maximum (FWHM) was employed to estimate the energy resolution (~0.64 eV). The inelastic background was subtracted using Smart background profile offered by Avantage software provided by the ThermoFisher Scientific and the observed XPS peaks were fitted by pseudo-Voight profiles.

For the thermal stability measurements, the sample, placed in the hot zone of the heating unit, was heated with a rate of ~ 3.6 K/min, and the XPS measurements were conducted with a 5 K step. The time of a single measurement (substrate signal, carbon signal) was ~40 seconds which limited possible temperature variation during the measurement. Additionally, to limit the potential irradiation-induced damage, especially at elevated temperatures[1], TP-XPS measurement were performed at the matrix of points evenly distributed over the area of ~6 mm^2. In that way, every temperature point was measured on the different sample spot. To exclude the influence of sample inhomogeneity on the results obtained, a calibration measurement was

performed before the TP-XPS experiments, in the course of which the substrate signal was measured in the points of the matrix used for thermal experiments afterwards (Figure S1). These measurements indicated excellent sample homogeneity within the studied area.

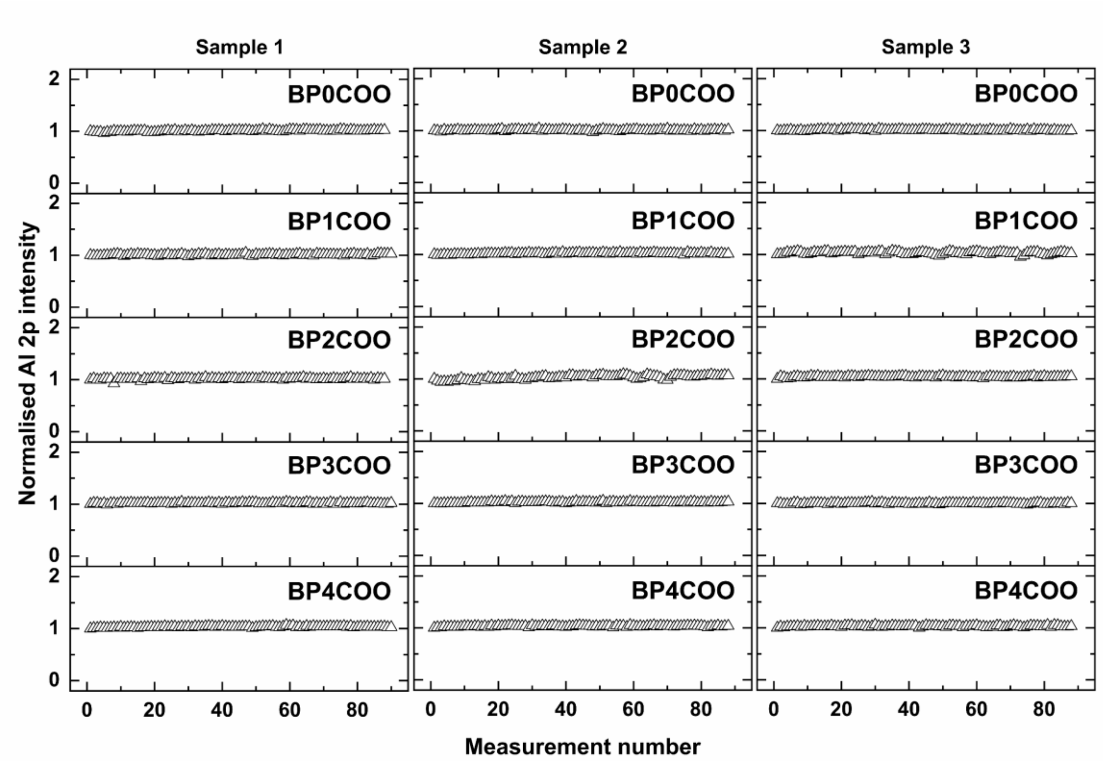

Figure S1. Calibration measurements, i.e., the Al 2p signal intensity measured in subsequent matrix points for all BPnCOO/AlO_x samples used in the thermal stability experiments.

1.3 **NEXAFS spectroscopy measurements.** C K-edge spectra were measured at the HE-SGM beamline of the synchrotron radiation facility BESSY II (Berlin, Germany) by partial yield detector at retarding voltage of -150 V with energy resolution ~0.3 eV. The photon energy scale was calibrated to a π* resonance of HOPG at 285.38 eV.[2] The samples were subjected to linearly polarized synchrotron light (polarization factor ~ 0.9), whose incidence angle was varied from 90° to 20° to monitor possible linear

dichroism reflecting the orientation of the molecular layers.[3] The spectra were normalized by the transmission function measured for freshly sputtered gold and are presented in the standard form with intensity set to zero at the pre-edge and the unity jump at the far post-edge region.

2. Details of the NEXAFS analysis

The intensity of a resonance associated with a vector-type molecular orbital could be expressed as:

$$(1)\ I(\alpha) = A\left\{P \times \frac{1}{3}\left[1 + \frac{1}{2}(3\,cos^2\alpha - 1)(3\,cos^2\rho - 1)\right] + (1 - P)\frac{1}{2}sin^2\rho\right\},$$

where A is an orbital-specific constant, α is an incidence angle of the X-ray with polarization factor P, and ρ is an angle between the π_1* resonance TDM and the substrate normal.[3] To exclude the A constant (unknown) from the evaluation procedure, the relative intensities - I(α)/I(20°), I(α)/I(30°), I(α)/I(55°), and I(α)/I(90°) - were used and the respective formulas were employed for the fitting procedure. The final ρ value for particular SAM is taken as the mean value over the above four fits.

3. TP-XPS measurements

3.1 Details of the TP-XPS analysis

To determine the desorption temperature for a particular sample, the intensity of the main C 1s peak was plotted as a function of T. The resulting profile was subsequently smoothed using a FFT filtering method. The minimum of the first derivative of the smoothed profile was taken as the sample desorption temperature. For each BPnCOO SAM, three samples were measured, and the arithmetic mean of three desorption

temperatures was calculated, giving the SAM desorption temperature (**Table 1**). The measurement uncertainty was estimated as the maximal difference between a single value in the series and the mean value.

3.2 Results of the TP-XPS measurements

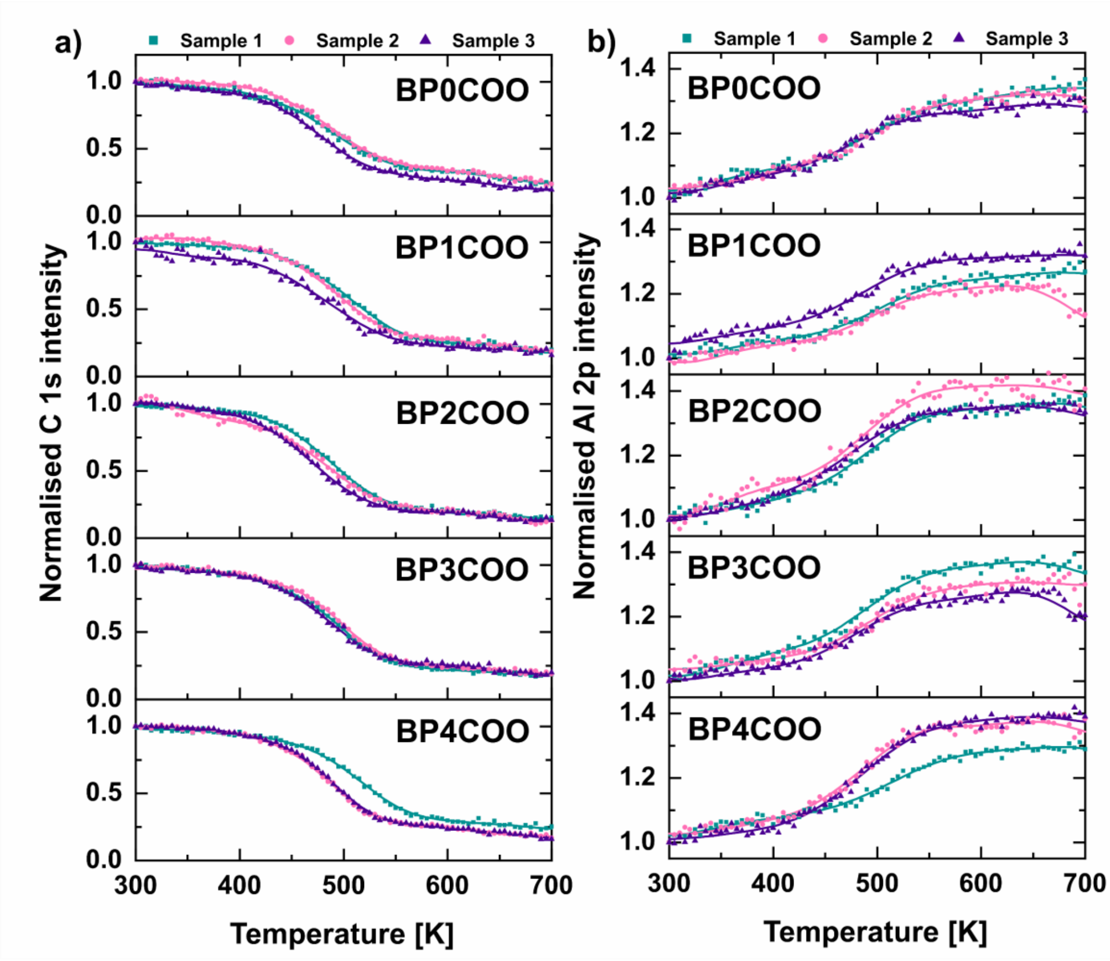

Figure S2. Normalized intensities of (a) the main C 1s peak and (b) total Al 2p signal as functions of temperature for all BPnCOO/AlO_x samples tested in the thermal stability experiments.

Table S1. Summary of the desorption temperatures obtained for the individual BPnCOO/AlO_x samples.

	Desorption temperature [K]		
	Sample 1	Sample 2	Sample 3
**BP0COO**	489	490	478
**BP1COO**	511	497	491
**BP2COO**	490	486	471
**BP3COO**	498	498	489
**BP4COO**	518	488	490

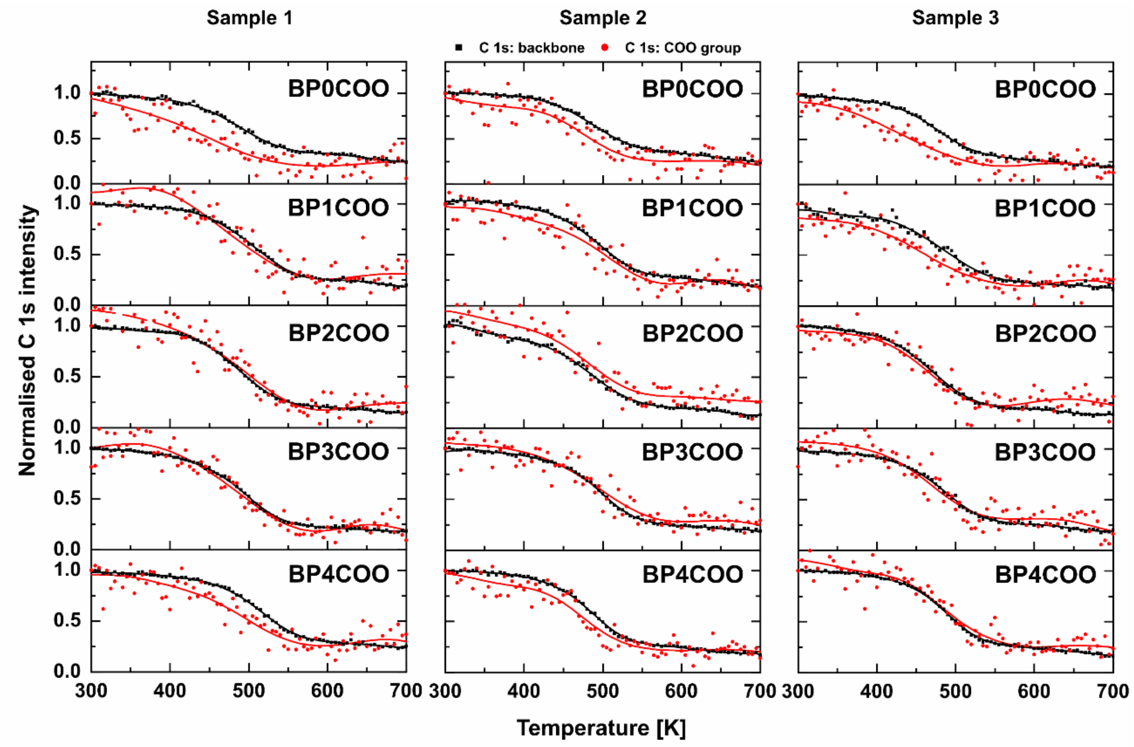

Figure S3. Normalized intensities of the C 1s peaks associated with the molecular backbone (black squares) and carboxylic group (red circles) as functions of temperature for all BPnCOO/AlO_x samples tested in the thermal stability experiments.

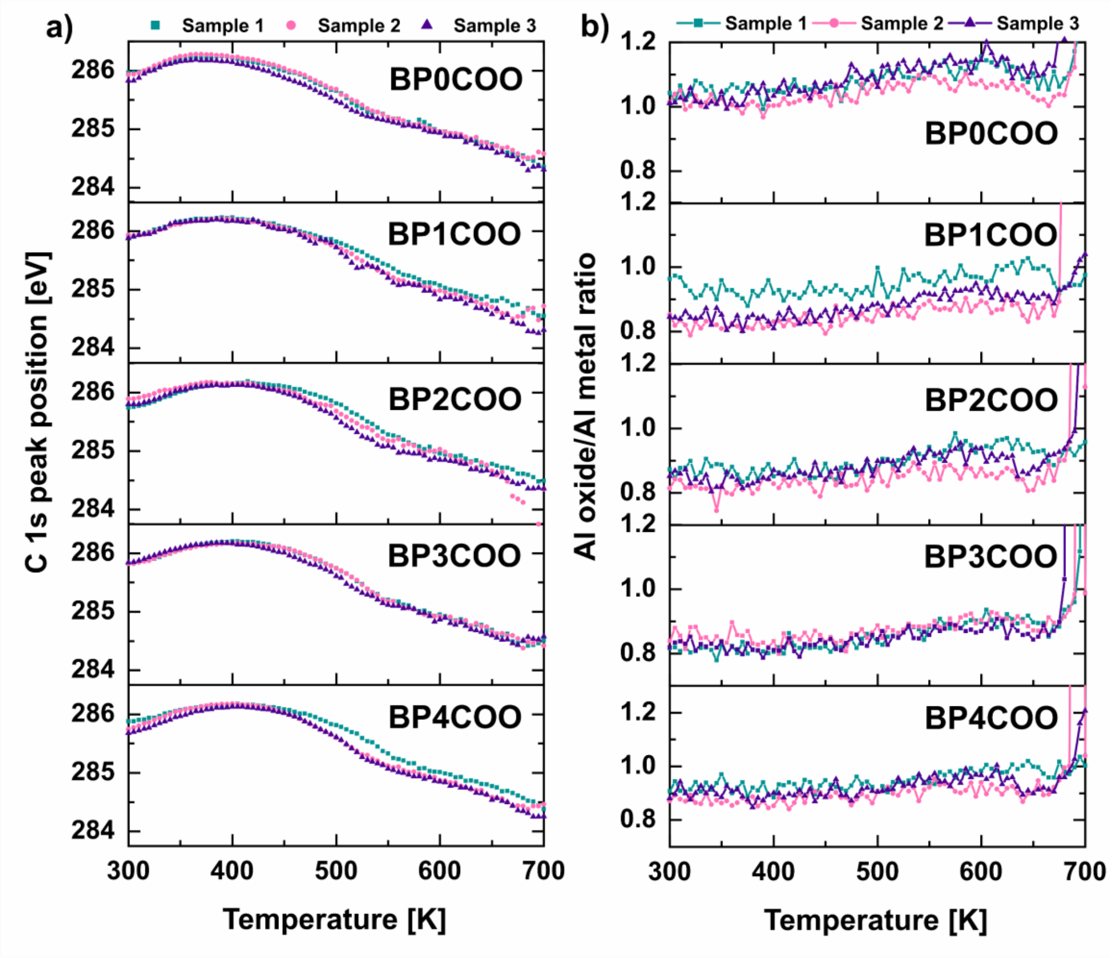

Figure S4. (a) The BE position of the main peak of C 1s peak (associated with the molecular backbone) and (b) the ratio between the metallic and oxide components of the Al 2p signal as functions of temperature for all BPnCOO/AlO_x samples used in the thermal stability experiments.

References

(1) Shaporenko, A.; Zharnikov, M.; Feulner, P.; Menzel, D. Quantitative Analysis of Temperature Effects in Radiation Damage of Thiolate-Based Self-Assembled Monolayers. *Journal of Physics: Condensed Matter* **2006**, *18* (30), S1677–S1689. https://doi.org/10.1088/0953-8984/18/30/S15.

(2) Batson, P. E. Carbon 1s Near-Edge-Absorption Fine Structure in Graphite. *Phys Rev B* **1993**, *48* (4), 2608–2610. https://doi.org/10.1103/PhysRevB.48.2608.

(3) Stöhr, J. *NEXAFS Spectroscopy*; Springer Berlin Heidelberg: Berlin, Heidelberg, 1992; Vol. 25. https://doi.org/10.1007/978-3-662-02853-7.
