# Supplementary material for: Odd–Even Effects in the Structure and Thermal Stability of Carboxylic Acid Anchored Monolayers on Naturally Oxidized Aluminum Surface
Source: J Phys Chem Lett. 2025 Mar 25;16(13):3343–50. doi: 10.1021/acs.jpclett.5c00500 (PMC11973916; doi:10.1021/acs.jpclett.5c00500)
Supplement: Supplementary file 2 — jz5c00500_si_002.pdf [file jz5c00500_si_002.pdf]

jz-2025-00500d.R1

Name: Peer Review Information for "Odd-Even Effects in the Structure and Thermal Stability of Carboxylic Acid Anchored Monolayers on Naturally Oxidized Aluminum Surface"

First Round of Reviewer Comments

Reviewer: 1

Comments to the Author

The manuscript with ID jz-2025-00500d by Cegiełka and colleagues reports an interesting study on the development of self-assembled monolayers on aluminium oxide surfaces and the generation of odd–even effects associated with the parity of building blocks forming the backbones of the molecular units of the SAMs. The study uses a combination of characterisation techniques (e.g., XPS, NEXAFS, thermogravimetry) to analyse a range of features of the monolayers (e.g., orientation, thickness, compactness). Several potential impacts of this technology are proposed but no practical application is demonstrated beyond the analysis of SAMs structure and thermal stability. This is reasonable considering the limited space. However, it is my opinion that to extrapolate the implications of this study to create an impact on applications will require further investigations. It would also be interesting to analyse if this technique can also be extended to other valve metals such as titanium and tantalum, which are also used in electronics and sensors, and which also form native oxide layers. On a minor note, the manuscript does not have sections headings, which makes the reading confusing (e.g., abstract, introduction, results and discussion).

Overall, I believe that the manuscript can be considered for publication in The Journal of Physical Chemistry Letters. However, I recommend a moderation of tone in the potential impact of this study on applications, unless the Authors provide more robust data on these (e.g., growth of organic semiconductors and electronic characterisation).

Author's Response to Peer Review Comments:

Prof. dr hab. Piotr Cyganik

Department of Physics of Nanostructures

and Nanotechnology

e-mail: piotr.cyganik@uj.edu.pl

tel. +48 (12) 663-58-38

Kraków 18.03.2025

Senior Editor,

The Journal of Physical Chemistry Letters

Dear Editor,

We would like to thank you and the Reviewer for the positive reception of our manuscript. In the following part of this letter, we provide our point-by-point replies to the Reviewer comments/suggestions and the note of the Editorial office.

We hope that the revised version of our manuscript meets the high standards of the *Journal of Physical Chemistry Letters.*

Sincerely yours,

Piotr Cyganik

Reply to the comments of Reviewer 1

Recommendation: *This paper is publishable subject to minor revisions noted. Further review is not needed.*

Comments:
The manuscript with ID jz-2025-00500d by Cegiełka and colleagues reports an interesting study on the development of self-assembled monolayers on aluminium oxide surfaces and the generation of odd–even effects associated with the parity of building blocks forming the backbones of the molecular units of the SAMs. The study uses a combination of characterisation techniques (e.g., XPS, NEXAFS, thermogravimetry) to analyse a range of features of the monolayers (e.g., orientation, thickness, compactness). Several potential impacts of this technology are proposed but no practical application is demonstrated beyond the analysis of SAMs structure and thermal stability. This is reasonable considering the limited space. However, it is my opinion that to extrapolate the implications of this study to create an impact on applications will require further investigations.

Author reply: We thank the Reviewer for the positive reception of our work. We fully agree that the application of hybrid aromatic-aliphatic SAMs will require further investigations which, however, are beyond the scope of our study dealing with fundamental aspects of molecular self-assembly on an application relevant substrate – well within the scope of the *J. Phys. Chem. Lett.* To this end, we intentionally use the wording "believe" in relation to possible applications, both in the Abstract and final statements.

In view of the reviewer comment, we have modified and extended these statements:

Before: "Thus, considering the high structural quality and thermal stability, we believe that biphenyl-substituted carboxylic acids on aluminum are promising systems for application in organic electronics."

Modification: "Thus, considering the high structural quality, tunable packing density, and thermal stability, we believe that biphenyl-substituted carboxylic acids on aluminum are promising systems for application in organic electronics. An additional option is the decoration of the BPnCOO molecules with specific tail groups, promoting the growth of an organic semiconductor and/or affecting positively the charge carrier density in the adjacent active layer, e.g. by mitigation of interfacial defects. Also, these molecules can be possibly applied to other application-relevant oxide substrates, including naturally oxidized titanium and tantalum. This, however, will require further investigations, which will be hopefully stimulated by the presented results."

It would also be interesting to analyse if this technique can also be extended to other valve metals such as titanium and tantalum, which are also used in electronics and sensors, and which also form native oxide layers.

Author reply: The aromatic SAMs with anchoring carboxylic groups can be formed on TiO_2 surface, as documented in literature including also our own former research (*J. Phys. Chem. C* 2009, *113,* 9309–9315, J. Phys. Chem. C 2011, *115*, 4134–4144). The Ta_2O_5 surface can also be modified by SAMs, with silane and phosphoric acid anchoring groups tried so far. We think, however, that, carboxylic group should also be suitable to this substrate.

In view of the reviewer comment, we included a note about titanium and tantalum into the modified manuscript (see the reply to the previous comment).

On a minor note, the manuscript does not have sections headings, which makes the reading confusing (e.g., abstract, introduction, results and discussion).

Author reply: The manuscript is prepared in accordance with the requirements of the *J. Phys. Chem. Lett.*, which exclude section headings.

Overall, I believe that the manuscript can be considered for publication in The Journal of Physical Chemistry Letters. However, I recommend a moderation of tone in the potential impact of this study on applications, unless the Authors provide more robust data on these (e.g., growth of organic semiconductors and electronic characterisation).

Author reply: We can only repeat that our study deals with fundamental aspects of molecular self-assembly on an application relevant substrate, letting a demonstration of specific applications for further investigations by us and the others. The wording "believe" in relation to possible applications is already sufficiently moderate in our opinion.

At the same time, as mentioned above, we modified and extended the conclusive statements of our manuscript, meeting the concerns by the reviewer.

Reply to the Editorial comments

1. Please include author names, article titles, journal name, publication year, and at least the first page number for the following incomplete journal reference: 69.

Author reply: the reference 69 has been completed

2. The TOC graphic should fit in an area no larger than 3.25 in. × 1.75 in. (approx. 8.25 cm × 4.45 cm) and should have adequate resolution and clarity. Confirm that all text is legible at this size.

Author reply: the TOC graphic has been readjusted in accordance with the requirements.
